# Protective Effect of Edaravone against Carbon Monoxide Induced Apoptosis in Rat Primary Cultured Astrocytes

**DOI:** 10.1155/2017/5839762

**Published:** 2017-02-02

**Authors:** Xiaodan Xu, Hong Zhang, Ke Wang, Tao Tu, Yuan Jiang

**Affiliations:** ^1^Department of Rehabilitation Medicine, The First Affiliated Hospital of Chengdu Medical College, Chengdu 610500, China; ^2^Department of Anesthesiology, The First Affiliated Hospital of Chengdu Medical College, Chengdu 610500, China

## Abstract

*Objective*. To observe the protective effect of edaravone (Eda) on astrocytes after prolonged exposure to carbon monoxide (CO) and further to investigate the potential mechanisms of Eda against CO-induced apoptosis.* Methods*. The rat primary cultured astrocytes were cultured in vitro and exposed to 1% CO for 24 h after being cultured with different concentrations of Eda. MTT assay was used to detect the cytotoxicity of CO. Flow cytometry was used to detect the apoptosis rate, membrane potential of mitochondria, and ROS level. The mRNA and protein expressions of Bcl-2, Bax, and caspase-3 were assessed by real-time PCR and Western blotting analysis, respectively.* Results*. Eda can significantly suppress cytotoxicity of CO, and it can significantly increase membrane potential of mitochondria and Bcl-2 expressions and significantly suppress the apoptosis rate, ROS level, Bax, and caspase-3 expressions.* Conclusion.* Eda protects against CO-induced apoptosis in rat primary cultured astrocytes through decreasing ROS production and subsequently inhibiting mitochondrial apoptosis pathway.

## 1. Introduction

Carbon monoxide (CO) is a tasteless, colorless, odorless, and nonirritating but highly toxic gas [1], and it is a by-product of the incomplete combustion of organic material, charcoal burning, domestic heating appliances and systems, and car exhaust. Acute CO poisoning is the most common type of poisoning worldwide, and it accounts for many deaths by accidental, suicide, or otherwise [2]. Inhaled CO mainly is bind with hemoglobin containing Fe^2+^ to form carboxyhemoglobin (COHb) [3], and CO has 200 times the affinity for hemoglobin than oxygen that cause impairs oxygen delivery and peripheral utilization leading to tissue hypoxia, oxidative stress, and inflammation within short period (hours or days) after CO poisoning [4, 5]. Thus, CO may cause neurological, cerebrovascular, or cardiovascular disorders, such as ischemia, encephalopathy, and peripheral nerve injury.

Most surviving patients can recover completely, but two neurological syndromes may occur in some: permanent neurological sequelae (PNS) and delayed neurological sequelae (DNS) [6]. About 40% of surviving patients develop DNS, also known as delayed encephalopathy after acute carbon monoxide poisoning (DEACMP), after a lucid interval from a few days up to months from the acute CO poisoning. The symptoms of DNS include impaired judgment, poor concentration and memory, cognitive dissonance, personality changes, psychosis, and even Parkinsonism symptoms. Unfortunately, the mechanisms of DNS in humans and in experimental animals remain unclear [7, 8]. Some previous studies suggested that DNS may be the outcome of acute CO poisoning induced cerebral cellular hypoxia, postischemic reperfusion injury, free radical damage, immune damage, and apoptosis [6]. Free radical damage has important role in postischemic reperfusion injury of cerebral cells after acute CO poisoning as high concentration of CO can be combined with the mitochondrial enzyme complex IV which cause accelerated mitochondrial respiratory chain electron leakage leading to excessive ROS production [9]. Astrocytes are the most abundant glial cell types in the brain and play an essential role in the antioxidant defense mechanisms of brain tissue. Astrocytes also can provide nutritional and metabolic support to neurons and regulate synaptic activity [10]. High amounts of exogenous CO causes oxidative stress and mitochondrial dysfunction, leading to astrocytic apoptosis and impairment in astrocyte function [11, 12]. Therefore, the inhibition of astrocytic apoptosis may offer protective effects on neurodegenerative disorders after acute CO poisoning.

Edaravone (Eda) is a novel synthetic small-molecule free radical scavenger [13]. It can protect brain tissue from damage and enhance mental work capacity, due to its high liposolubility and permeability to blood-brain barrier [14]. Thus, it is considered that Eda may be potentially useful for many brain diseases. Some scholars suggested that Eda plays a protective role in the cytotoxicity induced by nitric oxide [15], manganese [16], and neurotoxin MPP+ [13] in astrocytes. However, the protective effects of Eda against CO-induced cytotoxicity in astrocytes are yet to be further investigated. In this present study, we investigated the protective effects of Eda on the CO-induced cytotoxicity in rat primary cultured astrocytes and further explored the potential mechanism involved in the neuroprotective role of Eda.

## 2. Materials and Methods

### 2.1. Major Reagents

Edaravone injection was purchased from Jiangsu Simcere Pharmaceutical Co., Ltd. (molecular weight: 174.20). Fetal bovine serum (FBS), phosphate buffered solution (PBS), Dulbecco's modified Eagle's medium (DMEM), and trypsin (with phenol red) were purchased from Hyclone (GE Healthcare). MTT cell proliferation and cytotoxicity assay kit, Annexin V-FITC apoptosis detection kit, reactive oxygen species (ROS) assay kit, Rhodamine 123, Trizol, RIPA lysis buffer, enhanced BCA protein assay kit, and SDS-PAGE gel quick preparation kit were purchased from Beyotime. Revert aid first strand cDNA synthesis kit and Super Signal West Pico Chemiluminescent Substrate Trial kit were purchased from Thermo Fisher Scientific Inc. SYBR® Premix Ex Taq™ II was purchased from Takara Bio Inc. Bcl-2 antibodies, Bax antibodies, caspase-3 antibodies, and *β*-actin antibodies were purchased from Santa Cruz Biotech.

### 2.2. Cell Culture

The rat primary cultured astrocytes were prepared from cerebral cortex of newborn Sprague-Dawley (SD) rats as previously described [13], and all animal experimental protocol and care were approved by the Institutional Animal Care and Use Committee of Chengdu Medical College. Briefly, after newborn SD rats were killed by rapid decapitation, the cerebral cortices were removed and separated from meninges, blood vessels, and basal ganglia and cut to pieces. The pieces were dissociated with 0.25% trypsin and terminated by DMEM medium supplemented with 10% FBS, then centrifugated, and seeded on poly-lysine-coated cell culture flasks (Sigma). The cell culture flasks were placed in incubator at 37°C in a humidified atmosphere containing 5% CO_2_. After the cell culture flasks have been incubated for 24 h, the culture medium was replaced, and then changed every 2-3 days. The rat primary cultured astrocytes were identified by immunocytochemistry before experiments, and results have shown that more than 98% of the cells stained positively for the astrocytic marker glial fibrillary acid protein (Sigma).

### 2.3. Experimental Protocols

The rat primary cultured astrocytes were replated on poly-lysine-coated cell culture 96-well or 6-well plates and randomly divided into control group, CO group, 0.1 *μ*M Eda group, 1 *μ*M Eda group, 10 *μ*M Eda group, and 50 *μ*M Eda group. The astrocytes in CO group were exposed to carbon monoxide (the volume fraction is 1%) in a sealed chamber containing 5% CO_2_ for 24 h at 37°C. The 0.1 *μ*M Eda group, 1 *μ*M Eda group, 10 *μ*M Eda group, and 50 *μ*M Eda group were preexposed to different concentrations of Eda (0.1 *μ*M, 1 *μ*M, 10 *μ*M, and 50 *μ*M) for 2 h and then exposed to 1% CO in a sealed chamber containing 5% CO_2_ for 24 h at 37°C, respectively. The astrocytes in control group were placed in a sealed chamber containing 5% CO_2_ for 24 h at 37°C.

### 2.4. MTT Assay

Cytotoxicity of CO was assessed using the 3-(4,5-dimethylthiazol-2-yl)-2,5-diphenyl-tetrazolium bromide (MTT) reduction assay as previously described [17]. Briefly, the astrocytes of different group were seeded into 96-well for 24 h and then incubated with 200 *μ*L MTT-containing medium (0.5 mg/mL, diluted with fresh serum-free DMEM medium) for 4 h at 37°C after experimenting. Finally, the MTT-containing medium was removed and 150 *μ*L dimethyl sulfoxide (DMSO) was added to each well. The optical density (OD) was measured using an iEMS Analyzer at a wavelength of 570 nm. The cytotoxicity of CO was calculated using the following equation: cytotoxicity (%) = (OD_570_ control group − OD_570_ treatment group)/OD_570_ control group × 100.

### 2.5. Apoptotic Analysis

Alteration of cell apoptosis level was detected by flow cytometry (FCM) as previously described [17]. Briefly, after being exposed to CO for 24 h, the astrocytes of each group were suspended in the 195 *μ*L of 1x Annexin V-FITC binding buffer and incubated with 5 *μ*L of Annexin V-FITC at room temperature (RT) for 10 min; then the astrocytes were centrifuged at 1000 rpm for 5 min. Finally, the astrocytes were resuspended in 190 *μ*L of binding buffer and incubated with 10 *μ*L of propidium iodide (PI) solution at RT for 5 min. Cell apoptosis level was analyzed using FCM according to the manufacture's instruction.

### 2.6. Mitochondrial Membrane Functional Measurement

Mitochondrial membrane potential was measured using FCM with Rhodamine 123 staining as previously described [18]. Briefly, after being exposed to CO for 24 h, the astrocytes of each group were incubated with Rhodamine 123 (1 *μ*M) for 30 min at 37°C in the dark. The astrocytes were washed and resuspended in PBS and then analyzed using FCM.

### 2.7. ROS Measurement

Cellular ROS level was measured using FCM with DCFH-DA kit as previously described [18]. Briefly, after being exposed to CO for 24 h, the astrocytes of each group were incubated with DCFH-DA kit (10 *μ*M) for 20 min at 37°C in the dark. The astrocytes were washed and resuspended in PBS and then analyzed using FCM.

### 2.8. Real-Time PCR Analysis

The mRNA expressions of Bcl-2, Bax, and caspase-3 were analyzed by real-time PCR as previously described [19]. Briefly, after being exposed to CO for 24 h, the total RNA of astrocytes of each group were extracted using Trizol reagent according to the manufacturer's instructions and quantified by spectrophotometry at a wavelength of 260 nm. Reverse transcription actions and PCR were performed using reverse transcriptase, oligo (DT) primers, and Taq DNA polymerase. The primers used were as follows: Bcl-2 forward, 5′-TCCGCATCAGGAAGGCTAGA-3′ and reverse, 5′-AGGACCAGGCCTCCAAGCT-3′; Bax forward, 5′-CCTTTTCTACTTTGCCAGCAAAC-3′ and reverse, 5′-GAGGCCGTCCCAACCAC-3′; caspase-3 forward, 5′-AGTCTGACTGGAAAGCCGAA-3′ and reverse, 5′-CGGGATCTGTTTCTTTGCAT-3′; and GAPDH forward, 5′-CAGGAGGCATTGCTGATGAT-3′ and reverse, 5′-GAAGGCTGGGGCTCATTT-3′. The reaction was initiated with denaturation at 95°C for 30 sec, followed by 40 cycles of 95°C for 5 sec, 60°C for 60 sec (annealing), a terminal extension step at 95°C for 10 sec, and a final holding stage at 4°C. GAPDH was used as an internal control, and relative mRNA expressions were defined as the ratio of target genes expression to GAPDH expression.

### 2.9. Western Blot Analysis

The protein expressions of Bcl-2, Bax, and cleaved caspase-3 were analyzed by Western blot analysis as previously described [19]. Briefly, after being exposed to CO for 24 h, the total proteins of astrocytes of each group were extracted using RIPA lysis buffer. Protein samples were separated by sodium dodecyl sulfate-polyacrylamide gel electrophoresis and electrotransferred onto a polyvinylidene difluoride (PVDF) membrane. Then, the PVDF membrane was blocked for 2 h at RT in TBS-Tween 20 (TBST) buffer containing skimmed milk, washed with TBST three times, and incubated overnight at 4°C with 1/500 dilution of Bcl-2 antibodies, Bax antibodies, caspase-3 antibodies, and GAPDH antibodies, respectively. Subsequent to being washed with TBST three times, the PVDF membranes were incubated with horseradish peroxidase-labeled goat anti-rabbit immunoglobulin G (H + L) secondary antibody (1/4000 dilution) at 37°C for 1 h. Protein signals were detected using SuperSignal West Pico Chemiluminescent Substrate Trial kit and quantified by densitometry using Quantity One software (Bio-Rad).

### 2.10. Statistical Analysis

The statistical analysis was conducted using SPSS 20.0 for Windows software. Data were present as mean ± standard deviation (SD). Analysis of variance (ANOVA) was used to compare the differences between groups. Dunnett's T3 was used when the variances were unequal. *P* < 0.05 was considered to be statistical significance.

## 3. Results

### 3.1. MTT Assay

**Figure 1:**
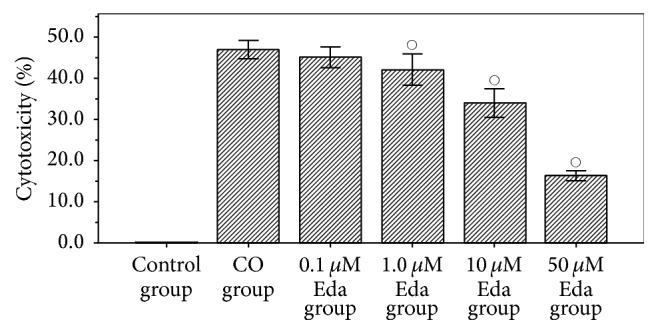
The results of cytotoxicity test. Cytotoxicity of each group was assessed using the MTT assay. Compared with CO group: °*P* < 0.01. Five biological replicates were prepared for each sample in this method.

After being preexposed to different concentrations of Eda (0 *μ*M, 0.1 *μ*M, 1 *μ*M, 10 *μ*M, and 50 *μ*M), the astrocytes were treated with CO for 24 h, and the cytotoxicity of CO was assessed using the MTT assay. As shown in Figure 1, the cytotoxicity of CO in CO group was 46.88 ± 1.79%, indicating that CO has significant direct cytotoxicity to astrocytes. However, the cytotoxicity of CO in 0.1 *μ*M Eda group, 1 *μ*M Eda group, 10 *μ*M Eda group, and 50 *μ*M Eda group was 45.02 ± 2.03%, 43.04 ± 3.04%, 33.92 ± 2.79%, and 16.31 ± 0.94%, respectively. By contrast, the cytotoxicity of CO in 1 *μ*M Eda group, 10 *μ*M Eda group, and 50 *μ*M Eda group was all significantly lower than that in CO group (all *P* < 0.01), and the cytotoxicity of CO decreases with drug concentration of Eda.

### 3.2. Apoptotic Analysis

**Figure 2:**
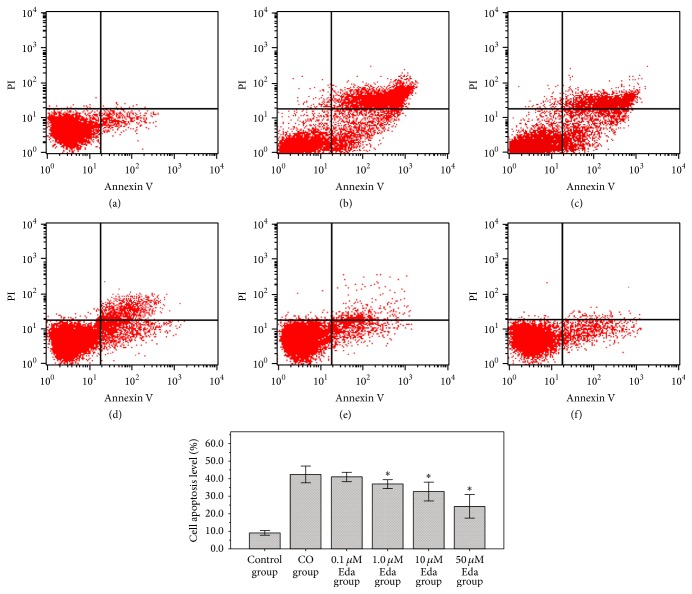
The results of apoptotic analysis. Cell apoptosis level of each group was detected by FCM. (a) Control group, (b) CO group, (c) 0.1 *μ*M Eda group, (d) 1.0 *μ*M Eda group, (e) 10 *μ*M Eda group, and (f) 50 *μ*M Eda group. Compared with CO group: ^*∗*^*P* < 0.01. Three biological replicates were prepared for each sample in this method.

After being preexposed to different concentrations of Eda (0 *μ*M, 0.1 *μ*M, 1 *μ*M, 10 *μ*M, and 50 *μ*M), the astrocytes were treated with CO for 24 h, and the alteration of cell apoptosis level was detected by FCM. The results of FCM have shown that (Figure 2), after being exposed to CO for 24 h, numerous apoptotic cells appeared in the astrocytes of CO group and the cell apoptosis level was 42.45 ± 2.36%. It indicted that prolonged exposure to CO could induce cell apoptosis in astrocytes by direct and strong cytotoxicity. By contrast, the cell apoptosis level of 0.1 *μ*M Eda group, 1 *μ*M Eda group, 10 *μ*M Eda group, and 50 *μ*M Eda group was 40.97 ± 1.32%, 36.85 ± 1.27%, 32.64 ± 2.68%, and 24.9 ± 3.36%, respectively. The results have shown that, after astrocytes are preexposed to different concentrations of Eda (0.1 *μ*M, 1 *μ*M, 10 *μ*M, and 50 *μ*M), the cytotoxicity activity of CO was decreased, and the cell apoptosis level was gradually declined with drug concentration of Eda, particularly in 1 *μ*M Eda group, 10 *μ*M Eda group, and 50 *μ*M Eda group. The cell apoptosis levels of those groups were all significantly lower than that in CO group (all *P* < 0.01).

### 3.3. Mitochondrial Membrane Functional Measurement

**Figure 3:**
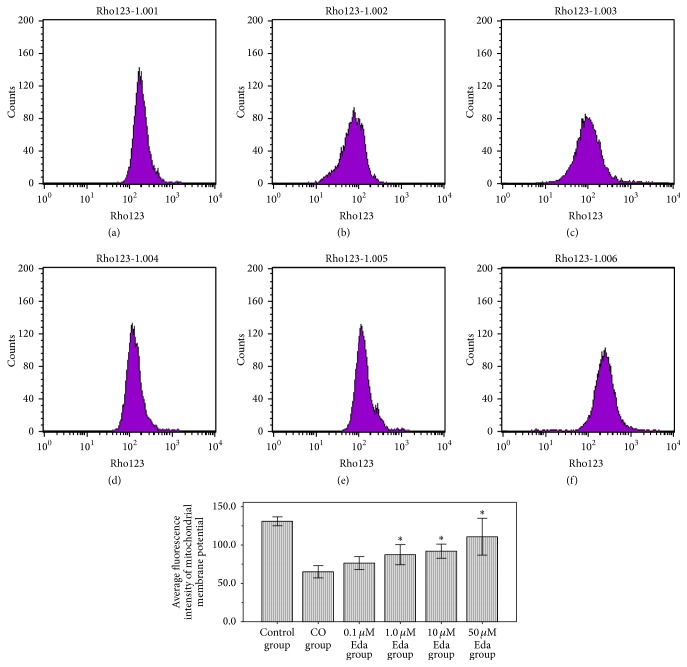
The results of mitochondrial membrane functional measurement. Mitochondrial membrane potential of each group was measured using FCM with Rhodamine 123 staining. (a) Control group, (b) CO group, (c) 0.1 *μ*M Eda group, (d) 1.0 *μ*M Eda group, (e) 10 *μ*M Eda group, and (f) 50 *μ*M Eda group. Compared with CO group: ^*∗*^*P* < 0.01. Three biological replicates were prepared for each sample in this method.

After being preexposed to different concentrations of Eda (0 *μ*M, 0.1 *μ*M, 1 *μ*M, 10 *μ*M, and 50 *μ*M),the astrocytes were treated with CO for 24 h, and the mitochondrial membrane potential was measured using FCM with Rhodamine 123 staining. The results have shown that (Figure 3) the mitochondrial membrane potential of CO group was significantly lower than control group, indicating that mitochondrial membrane depolarization occurred in the astrocytes treated with CO. Compared with the CO group, the mitochondrial membrane potential of 1 *μ*M Eda group, 10 *μ*M Eda group, and 50 *μ*M Eda group was significantly increased (all *P* < 0.01), and the mitochondrial membrane potential increases with drug concentration of Eda.

### 3.4. ROS Measurement

**Figure 4:**
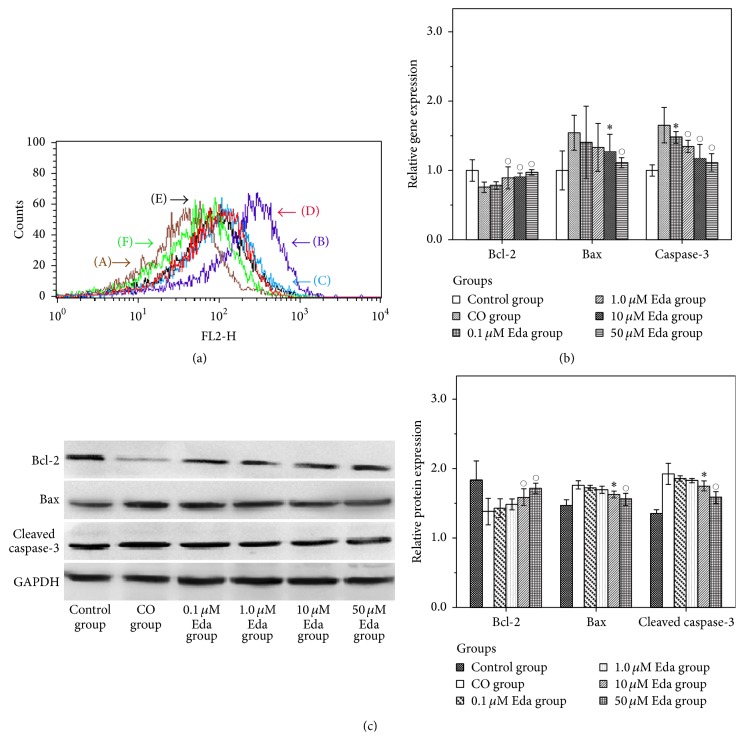
The results of ROS measurement, real-time PCR analysis, and Western blot analysis. (a) Cellular ROS level of each group was measured using FCM with DCFH-DA kit. (A) Control group, (B) CO group, (C) 0.1 *μ*M Eda group, (D) 1.0 *μ*M Eda group, (E) 10 *μ*M Eda group, and (F) 50 *μ*M Eda group. (b) The relative mRNA expressions of Bcl-2, Bax, and cleaved caspase-3 were estimated using real-time PCR analysis. Compared with CO group: ^*∗*^*P* < 0.05, °*P* < 0.01. (c) The protein expressions of Bcl-2, Bax, and caspase-3 were estimated using Western blot analysis. Compared with CO group: ^*∗*^*P* < 0.05, °*P* < 0.01. For each method, three biological replicates were prepared for each sample.

After being preexposed to different concentrations of Eda (0 *μ*M, 0.1 *μ*M, 1 *μ*M, 10 *μ*M, and 50 *μ*M), the astrocytes were treated with CO for 24 h, and the cellular ROS level was measured using FCM with DCFH-DA kit. As shown in Figure 4(a), in contrast with control group, the fluorescence peak of CO group was shifted to the right (Figure 4(a)(B)), indicating that the ROS level in the astrocytes after being exposed to CO for 24 h was markedly higher than the controlled astrocytes, and prolonged exposure to CO induced a large generation of ROS. However, after being preexposed to different concentrations of Eda, the ROS levels in the astrocytes treated with CO for 24 h were decreased with different degrees.

### 3.5. Real-Time PCR Analysis

The relative mRNA expressions of target genes were estimated using real-time PCR analysis. As shown in Figure 4(b), in contrast with control group, the mRNA expression of Bcl-2 was significantly decreased (*P* < 0.01) and the mRNA expressions of Bax and caspase-3 were significantly increased (all *P* < 0.01) in CO group. By contrast, after being preexposed to different concentrations of Eda, the mRNA expressions of Bcl-2 were increased and the mRNA expressions of Bax and caspase-3 were decreased with different degrees in the astrocytes treated with CO for 24 h.

### 3.6. Western Blot Analysis

The protein expressions of target genes were estimated using Western blot analysis. As shown in Figure 4(c), the protein expression of Bcl-2 was significantly decreased (*P* < 0.01) and the protein expressions of Bax and cleaved caspase-3 were significantly increased (all *P* < 0.01) in CO group compared with control group. In contrast with CO group, after being preexposed to different concentrations of Eda, the protein expressions of Bcl-2 were increased and the protein expressions of Bax and cleaved caspase-3 were decreased with different degrees in the astrocytes treated with CO for 24 h.

## 4. Discussion

CO poisoning is one of the common clinical emergencies. Once our bodies had inhaled CO, hemoglobin containing Fe^2+^ were bound with CO to form COHb, leading to oxygen deprivation, in particular, brain tissue hypoxia. Irreversible injury of brain neurons results from the context of prolonged exposure to CO [20]. DNS is a kind of delayed encephalopathy symptoms that occur in surviving patients after acute CO poisoning for a period of days to weeks [6]. In the period of time from before DNS occur in surviving patients, the clinical symptoms seem to be cured by effective symptomatic treatment, but DNS may occur after inadequate continuous treatment. Therefore, to investigate the mechanisms of DNS is greatly significant for effective prevention and treatment of DNS in clinic. Recent studies showed that cerebral cellular hypoxia, postischemic reperfusion injury, free radical damage, immune damage, and apoptosis may involve in the mechanisms of DNS [6], and it was considered that free radical damage and apoptosis were presumed to be one of the main pathogenesis. Astrocytes are important for the survival of neurons and defense effect of the central nervous system and play an important role in the process of scavenging oxygen free radicals in brain tissue [21]. Astrocytic apoptosis and dysfunction of astrocytes can negatively affect neuronal survival and may contribute to the pathogenesis of many brain diseases. In the present study, the results of MTT assay showed that the cytotoxicity of CO induces the death of astrocytes. Of course, CO can suppress the activity of succinate dehydrogenase which combined with mitochondrial inner membrane to partly affect the results of OD in MTT assay. However, it indirectly indicates that CO can damage mitochondrial respiratory chain function through inactivation of succinate dehydrogenase and ultimately lead to astrocytes death. Moreover, the results of apoptotic analysis showed that apoptosis was a main death mode of astrocytes after prolonged exposure to CO. The results of ROS and mitochondrial membrane potential measurement showed that vast ROS were generated in the astrocytes and causing mitochondrial membrane potential significantly decreased after a prolonged exposure to CO. Bcl-2 is a tumor suppressing gene, regulating mitochondrial membrane permeability to inhibit the signal transduction pathway of cell apoptosis. Proapoptotic gene Bax can accelerate irreversible apoptosis through disruption of mitochondrial membrane potential and activation of caspase-3. In the present study, we found that the mRNA and protein expressions of Bcl-2 were significantly decreased, and the mRNA and protein expressions of Bax and caspase-3 were significantly increased in the astrocytes after a prolonged exposure to CO. So we suspected that CO can induce the damage of mitochondrial membrane by causing the collapse of mitochondrial membrane potential and the generation of ROS that is causing mitochondrial apoptosis pathway activation in cerebral cells and ultimately lead to DNS in acute carbon monoxide poisoning patients. However, of course, CO has dual effect on modulation of apoptosis in astrocytes depending on its concentration. Almeida et al. [22] suggested that low concentrations of CO partially inhibited oxidative stress-induced apoptosis in astrocytes and played a positive role of protection by preventing mitochondrial potential depolarization and plasmatic membrane permeability, increasing the expressions of Bcl-2 and suppressing caspase-3 activation. Moreover, CO-induced ROS can become toxic or signaling depending on its concentration as well. Therefore, future investigation of the potential mechanism of different concentrations of CO in modulating mitochondrial function is necessary to be conducted.

Despite the acute carbon monoxide poisoning patients can receive various forms of treatments in present, hyperbaric oxygen therapy (HBOT) is the first choice and performing HBOT as early as possible is preferred, and postoperative prognosis is good. However, the preventive effects of HBOT on DNS remain controversial [23, 24]. Eda is a free radical scavenger, and it can effectively improve the tolerance of cerebral cells to hypoxia and protect the function of brain in the patients with acute CO poisoning. Thus, Eda can act as a potential therapy for preventing DNS. Li et al. [25] suggested that Eda attenuates brain damage in rats after acute CO poisoning by inhibiting oxidative stress and neuronal apoptosis. However, the protective mechanism of Eda against CO-induced cytotoxicity in astrocytes remains unclear. In the present study, after being preexposed to different concentrations of Eda (0.1 *μ*M, 1 *μ*M, 10 *μ*M, and 50 *μ*M), the cytotoxicity of CO and the apoptosis level gradually decline with drug concentration of Eda in astrocytes, indicating that Eda can effectively resist the cell damage caused by prolonged exposure to CO and has a protective effect on astrocytes. The results of ROS and mitochondrial membrane potential measurement showed that Eda can effectively eliminate ROS and attenuate the damage of mitochondrial membrane in astrocytes. Moreover, the results of mRNA and protein expressions of Bcl-2, Bax, and caspase-3 were indicating that Eda inhibited mitochondrial apoptosis pathway activation by increasing the expressions of Bcl-2 and suppressing the expressions of Bax and caspase-3. Therefore, we suggested that the protective mechanism of Eda may contribute to inhibiting mitochondrial apoptosis pathway activation by effectively scavenging ROS and protecting the function of mitochondria.

In conclusion, this study firstly reveals that Eda protects against CO-induced apoptosis in rat primary cultured astrocytes through decreasing ROS production and subsequently inhibiting mitochondrial apoptosis pathway. Eda appears to be effective for the treatment of patients with acute CO poisoning, and it can be useful in the prevention and treatment of DNS.

## Abbreviations

Eda: Edaravone
CO: Carbon monoxide
MTT: 3-(4,5-Dimethylthiazol-2-yl)-2,5-diphenyl-tetrazolium bromide
COHb: Carboxyhemoglobin
PNS: Permanent neurological sequelae
DEACMP: Acute carbon monoxide poisoning
DNS: Delayed neurological sequelae
ROS: Reactive oxygen species
FBS: Fetal bovine serum
PBS: Phosphate buffered solution
DMEM: Dulbecco's modified Eagle's medium
SD: Sprague-Dawley
DMSO: Dimethyl sulfoxide
OD: Optical density
FCM: Flow cytometry
RT: Room temperature
PI: Propidium iodide
PVDF: Polyvinylidene difluoride
HBOT: Hyperbaric oxygen therapy.
